# Evaluation of Medical College Departments of Ophthalmology in India

**DOI:** 10.4103/0301-4738.58483

**Published:** 2010

**Authors:** Sohan Singh Hayreh

**Affiliations:** Department of Ophthalmology & Visual Sciences, University of Iowa, Iowa City, USA

Dear Editor,

I was interested to read comments by Gopal[1] about “Evaluation of medical college departments of ophthalmology in India”, in the March-April 2009 issue. He rightly points out the roles of: (i) trainer, (ii) trainee, and (iii) equipment in the quality of training of ophthalmologists in India. I spent the first 34 years of my life in India - six of those as a faculty in a medical college, and have visited India from time to time since 1961 when I left India. Since leaving India, I have been on the medical faculties of the University of London, University of Edinburgh in Scotland, and now in the University of Iowa in the United States since 1973. Based on all this experience in India and abroad, I feel Gopal missed one important basic factor responsible for the problem: that is, to be appointed as either trainer or trainee in India, it is not uncommon to find that what matters most is “not what you know but whom you know”. Unless and until that basic problem is resolved, and merit is the only standard for appointment, it seems hard to envision much improvement.
